# MicroRNA-101 is repressed by EZH2 and its restoration inhibits tumorigenic features in embryonal rhabdomyosarcoma

**DOI:** 10.1186/s13148-015-0107-z

**Published:** 2015-08-06

**Authors:** Serena Vella, Silvia Pomella, Pier Paolo Leoncini, Marta Colletti, Beatrice Conti, Victor E. Marquez, Antonio Strillacci, Josep Roma, Soledad Gallego, Giuseppe M. Milano, Maurizio C. Capogrossi, Alice Bertaina, Roberta Ciarapica, Rossella Rota

**Affiliations:** Department of Oncohematology, Laboratory of Angiogenesis, Ospedale Pediatrico Bambino Gesù, IRCCS, Piazza S. Onofrio 4, 00165 Rome, Italy; Laboratorio di Patologia Vascolare, Istituto Dermopatico dell’Immacolata, IRCCS, Rome, Italy; Chemical Biology Laboratory, Frederick National Laboratory for Cancer Research, CCR, National Cancer Institute, NIH, Frederick, MD USA; Department of Biological, Geological and Environmental Sciences, Biology Unit, University of Bologna, Bologna, Italy; Laboratory of Translational Research in Paediatric Cancer, Vall d‘Hebron Research Institute and Vall d’Hebron Hospital, Universitat Autònoma de Barcelona, Barcelona, Spain; Department of Oncohematology, Clinical Unit, Ospedale Pediatrico Bambino Gesù, IRCCS, Piazza S. Onofrio 4, 00165 Rome, Italy

**Keywords:** MiR-101, EZH2, Histone methyltransferases, Polycomb proteins, Rhabdomyosarcoma, Cell motility, Cell proliferation, Anchorage-independent growth, Chromatin immunoprecipitation

## Abstract

**Background:**

Rhabdomyosarcoma (RMS) is a pediatric soft tissue sarcoma arising from myogenic precursors that have lost their capability to differentiate into skeletal muscle. The polycomb-group protein EZH2 is a Lys27 histone H3 methyltransferase that regulates the balance between cell proliferation and differentiation by epigenetically silencing muscle-specific genes. EZH2 is often over-expressed in several human cancers acting as an oncogene. We previously reported that EZH2 inhibition induces cell cycle arrest followed by myogenic differentiation of RMS cells of the embryonal subtype (eRMS). MiR-101 is a microRNA involved in a negative feedback circuit with EZH2 in different normal and tumor tissues. To that, miR-101 can behave as a tumor suppressor in several cancers by repressing EZH2 expression. We, therefore, evaluated whether miR-101 is de-regulated in eRMS and investigated its interplaying with EZH2 as well as its role in the *in vitro* tumorigenic potential of these tumor cells.

**Results:**

Herein, we report that miR-101 is down-regulated in eRMS patients and in tumor cell lines compared to their controls showing an inverse pattern of expression with EZH2. We also show that miR-101 is up-regulated in eRMS cells following both genetic and pharmacological inhibition of EZH2. In turn, miR-101 forced expression reduces EZH2 levels as well as restrains the migratory potential of eRMS cells and impairs their clonogenic and anchorage-independent growth capabilities. Finally, EZH2 recruitment to regulatory region of *miR-101-2* gene decreases in EZH2-silenced eRMS cells. This phenomenon is associated to reduced H3K27me3 levels at the same regulatory locus, indicating that EZH2 directly targets miR-101 for repression in eRMS cells.

**Conclusions:**

Altogether, our data show that, in human eRMS, miR-101 is involved in a negative feedback loop with EZH2, whose targeting has been previously shown to halt eRMS tumorigenicity. They also demonstrate that the re-induction of miR-101 hampers the tumor features of eRMS cells. In this scenario, epigenetic dysregulations confirm their crucial role in the pathogenesis of this soft tissue sarcoma.

**Electronic supplementary material:**

The online version of this article (doi:10.1186/s13148-015-0107-z) contains supplementary material, which is available to authorized users.

## Background

Rhabdomyosarcoma (RMS) is a soft tissue sarcoma that accounts for 50 % of all soft tissue sarcomas in childhood. Two major histological RMS subtypes have been identified, embryonal RMS (eRMS) and alveolar RMS (aRMS) [1]. eRMS is the most frequent form (about 70–80 %). RMS is believed to originate from immature skeletal muscle cells that are unable to differentiate [2]. Consistently, the induction of differentiation is considered of therapeutic value [3, 4]. Our and other groups have demonstrated that the histone methyltransferase polycomb-group (PcG) protein enhancer of zeste homologue 2 (EZH2) plays an important role in embryonal RMS tumorigenesis. EZH2 is the catalytic subunit of the polycomb repressor complex 2 (PRC2) that, through trimethylation of lysine 27 on histone H3 (H3K27me3), represses the transcription of specific target genes, thus preventing cell differentiation while promoting proliferation. As a matter of fact, EZH2 inhibits skeletal muscle differentiation by preventing the expression of miR-214 [5] while in turn, during myogenesis, miR-214 directly targets EZH2 3′UTR for degradation [6]. Regulatory feedback loops among EZH2 and microRNAs have been identified among the mechanisms by which EZH2 might sustain human tumorigenesis (for a review, see Ref. [7]). In line with this evidence, miR-214 is under-expressed in eRMS and its re-induction leads to myogenic differentiation [8]. Concordantly, we and others recently reported that EZH2 is markedly expressed in RMS primary specimens and cell lines compared to their normal counterparts [9, 10] and that inhibition of EZH2 represents a promising pro-differentiation therapeutic strategy in eRMS [11]. MiR-101 is a microRNA involved in a feedback loop with EZH2 [12, 13]. In the last few years, many studies have shown that miR-101 levels are decreased in several tumors, including breast, lung, prostate, ovarian, colon, and liver cancers, and that often miR-101 exerts a tumor suppressive role [14–17]. Recently, miR-101 has been shown to be induced during human myoblast differentiation [18]. In the present work, since EZH2 is abnormally up-regulated in eRMS, we sought to evaluate whether miR-101 might be altered in this tumor. Our results indicate that miR-101 is down-regulated in eRMS primary samples and cell lines, and knockdown or pharmacological inhibition of EZH2 up-regulates its levels. The restoration of miR-101 expression is able to reduce proliferation and migration rates and to hamper both the clonogenic and anchorage-independent capabilities of eRMS tumor cells. Moreover, our data also demonstrate that EZH2 inhibits miR-101 expression in eRMS cells by direct gene targeting. Altogether, these results suggest a negative feedback loop between miR-101 and EZH2 in eRMS cells and point on miR-101 as a potential anticancer microRNA.

## Results

### Inhibition of EZH2 restores miR-101 expression in embryonal RMS

**Fig. 1:**
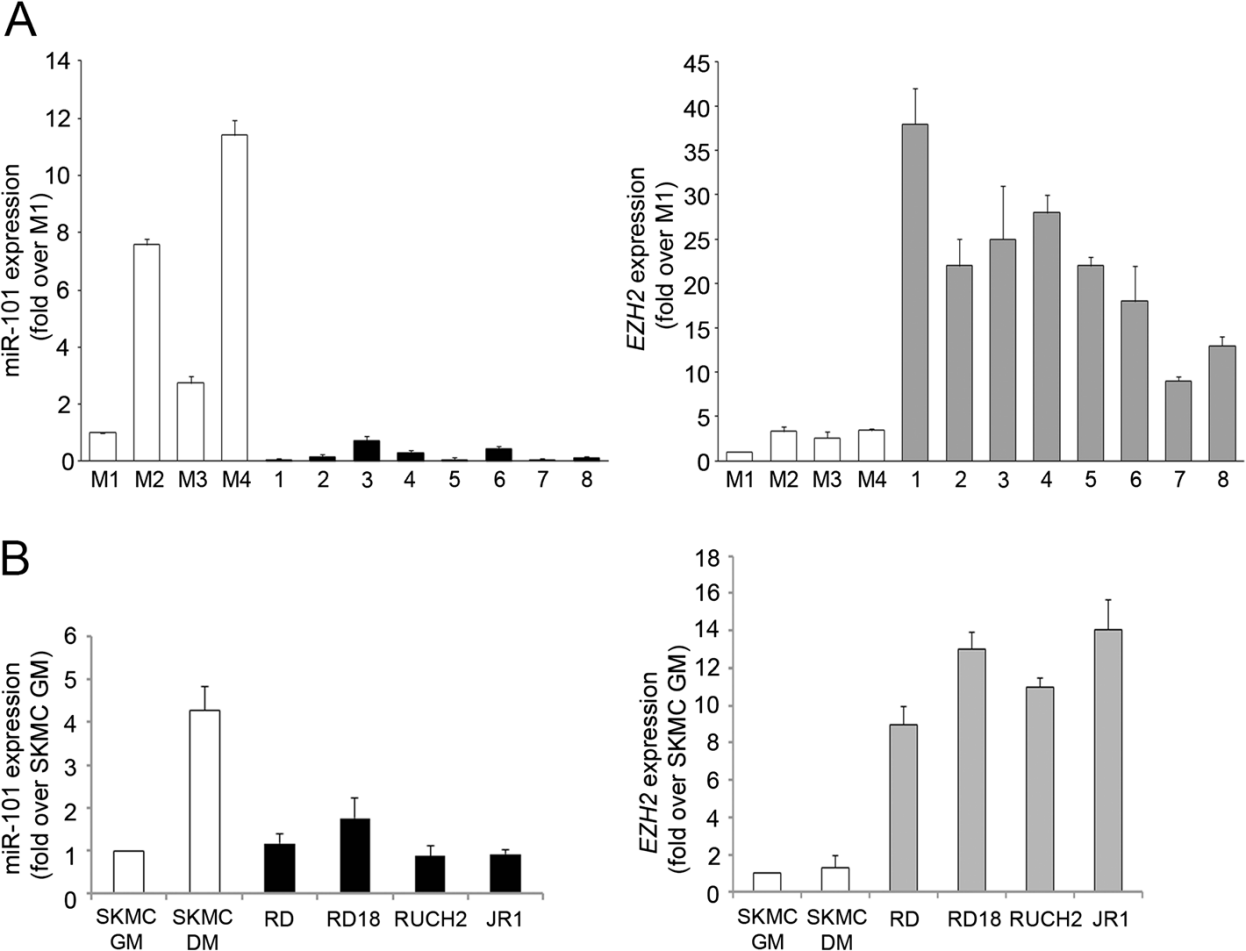
MiR-101 and EZH2 levels are inversely expressed in embryonal rhabdomyosarcoma (RMS) patients and cell lines compared to their controls. **a** Levels of mature miR-101 (*left panel*) and EZH2 (*right panel*) were determined by RT-qPCR in primary embryonal rhabdomyosarcoma (eRMS) samples (*black* and *grey bars*, respectively) and in normal skeletal muscles (M1-4) used as control tissues (*white bars*). Values normalized to snoU6 or GAPDH levels (respectively) were expressed as fold increase over M1 control tissue (1 arbitrary unit). **b** RT-qPCR of miR-101 (*left panel*) and EZH2 (*right panel*) in eRMS cell lines (RD, RD18, RUCH2, and JR1; *black* and *grey bars*, respectively) and normal skeletal muscle cells (SKMC) cultured in either growth medium (GM) or differentiating medium (DM) (as described in “Methods” section) were normalized to snoU6 or GAPDH levels, respectively, and were expressed as fold increase over SKMC GM cells (1 arbitrary unit). Two independent measurements were done in duplicate

To ascertain whether miR-101 expression is compromised in eRMS, we measured its levels along with those of EZH2 in primary tumors. We noticed that miR-101 was expressed at very low levels in eRMS primary samples compared to normal muscle tissues as controls (mean values: 0.23 ± 0.24 vs 5.7 ± 4.7, respectively) (Fig. 1a, left). Conversely, in line with previous reports [9, 10], EZH2 transcripts were markedly higher in the same group of primary samples compared to controls (mean values: 21.25 ± 8.86 vs 2.87 ± 1.31, respectively) (Fig. 1a, right). Similarly, miR-101 expression was lower in four eRMS cell lines (RD, RD18, JR1, RUCH2) than in differentiated human skeletal muscle cells (SKMC DM) (mean values 1.26 ± 0.49 vs 4.29 ± 0.55, respectively), instead being comparable to the level of miR-101 in proliferating skeletal myoblasts (SKMC GM) (Fig. 1b, left). Moreover, EZH2 mRNA levels were 11.76 ± 2.23 higher in the eRMS cell lines tested compared to SKMC (Fig. 1b, right).

**Fig. 2:**
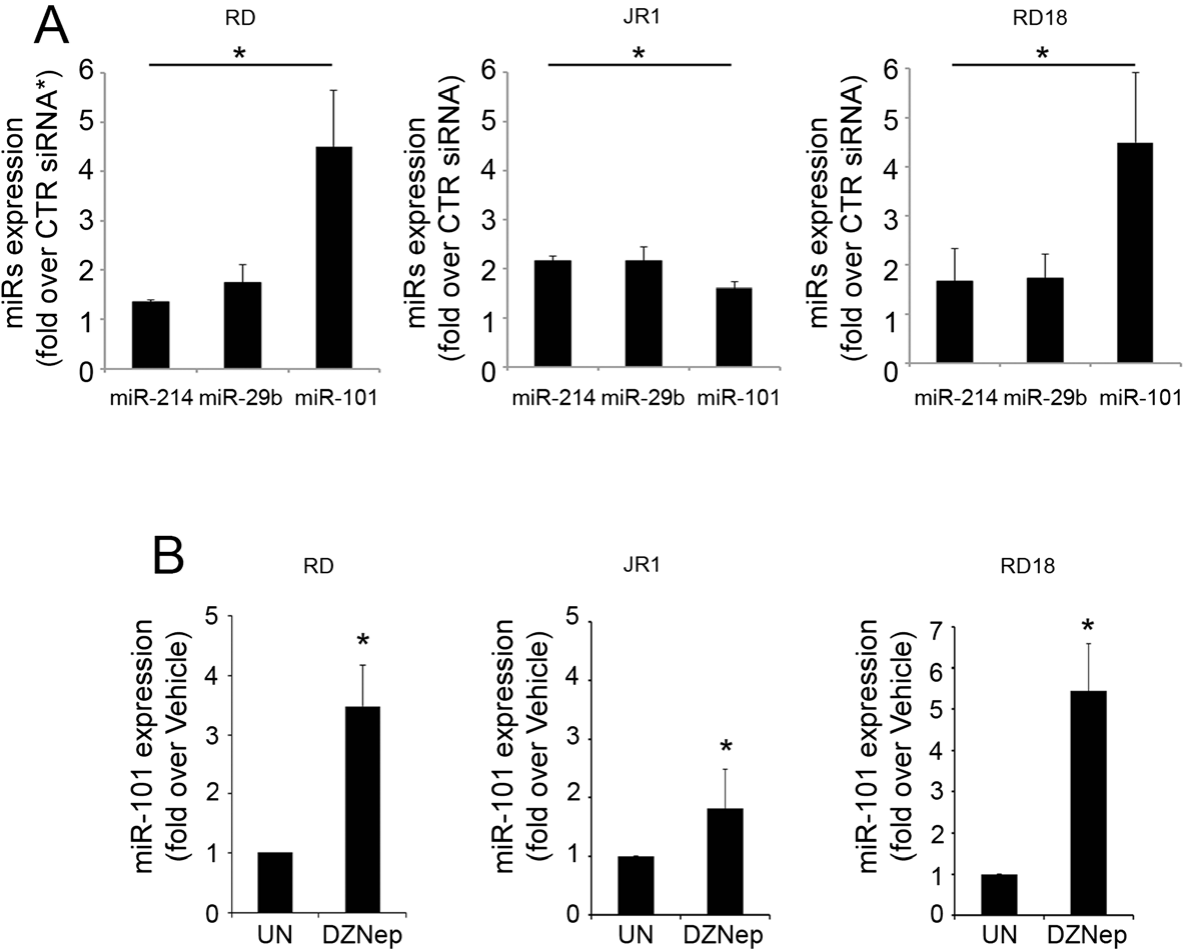
Inhibition of EZH2 restores endogenous miR-101 levels in eRMS cells. **a** RT-qPCR analysis of mature forms of miR-214, miR-29b, and miR-101 in RD, JR1, and RD18 cells 72 h post EZH2 siRNA transfection (RD were transfected with SMART pool siRNA EZH2 (*asterisks*), JR1 and RD18 were transfected with siRNA targeting 5′-UTR of EZH2, see “Methods” section). Data normalized using snoU6 and expressed as fold increase over a non-targeting siRNA (CTR siRNA, 1 arbitrary unit). *Columns*, means; *bars*, SD. Results from three independent experiments are shown. *P < 0.05 (Student’s *t*-test). **b** MiR-101 level in RD, JR1, and RD18 cells daily treated for 72 h with either S-adenosyl-L-homocysteine hydrolase inhibitor 3-deazaneplanocin A (DZNep) (5 μM) or vehicle (i.e., water, referred as untreated condition: UN), normalized using snoU6 and expressed as fold increase over UN (1 arbitrary unit). *Columns*, means; *bars*, SD. Results from three independent experiments are shown. *P < 0.05 (Student’s *t*-test)

To analyze whether miR-101 expression was affected by EZH2 modulation in eRMS, RD, JR1, and RD18 cell lines were silenced for EZH2 using either a pool of oligo siRNAs or oligo siRNA targeting the 5′UTR region of EZH2 mRNA, both previously validated (Additional file 1: Figures S1A and S1B) [11], and the expression of miR-101 together with that of other microRNAs known to be modulated by EZH2 in RMS, such as miR-214 and miR-29b [3, 8], was evaluated. As shown in Fig. 2a, EZH2 knockdown in eRMS cells increases the expression of miR-29b, miR-214, and miR-101 as soon as 72 h after siRNA transfection. Interestingly, miR-101 was the most up-regulated in RD and RD18 cells, showing an about 4-fold increase compared with cells transfected with a control non-targeting siRNA (CTR siRNA). Similarly, treatment with DZNep, the prototype of EZH2 inhibitors [11] which induces EZH2 degradation (Additional file 1: Figure S1C), resulted in the up-regulation of miR-101 with respect to cells treated with vehicle (about 3.5-, 1.5-, and 5-fold increase in RD, JR1, and RD18, respectively) (Fig. 2b). These results suggest that miR-101 and EZH2 are inversely expressed in eRMS and indicate EZH2 as a repressor of miR-101 in eRMS cells.

### Over-expression of miR-101 restrains the proliferation rate of embryonal RMS cells and reduces the endogenous levels of EZH2

**Fig. 3:**
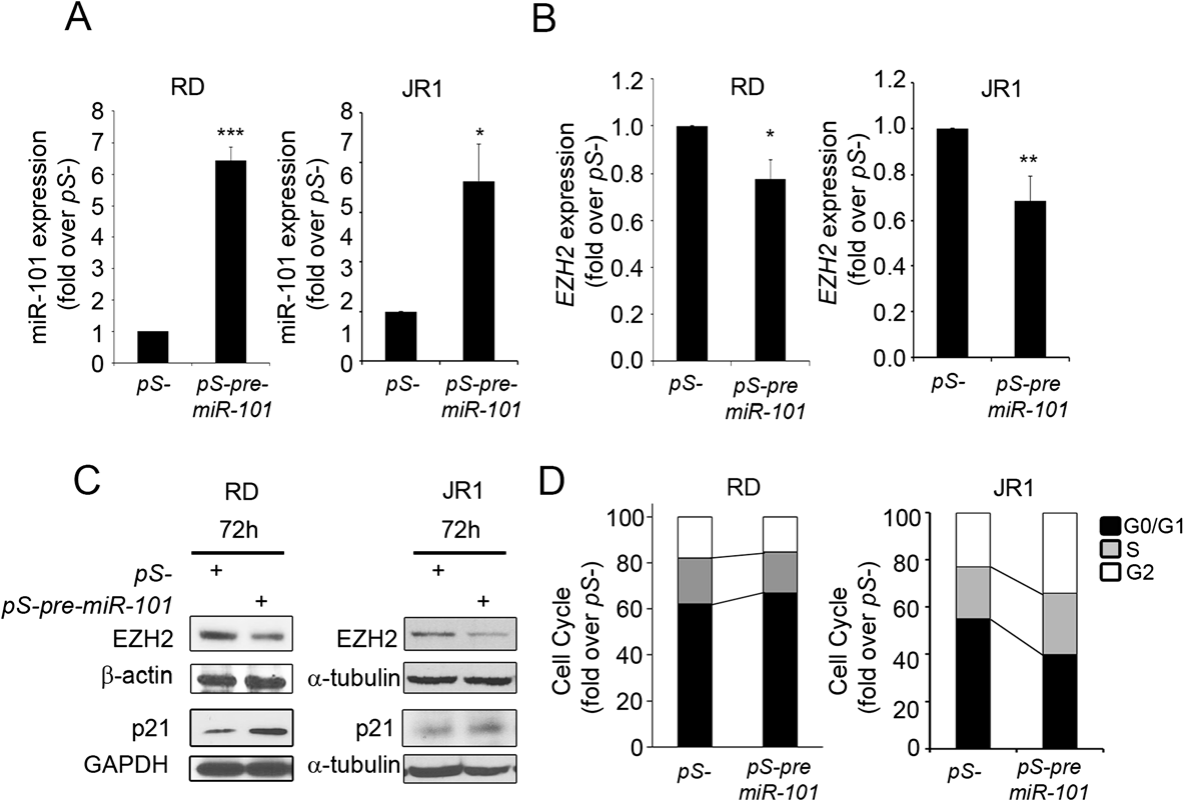
MiR-101 over-expression reduces EZH2 levels and cell proliferation in eRMS cells. RT-qPCR analysis of mature **a** miR-101 and **b** EZH2 in RD and JR1 cells 72 h post infection with pS-pre-miR-101 or control pS- retrovirus. Data were normalized using snoU6 and GAPDH levels respectively and expressed as fold increase over control (pS-, 1 arbitrary unit). *Columns*, means; *bars*, SD. Results from three independent experiments are shown. *P < 0.05, **P < 0.01, and ***P < 0.001 (Student’s *t*-test). **c** Western blots showing EZH2 and p21^Cip1^ levels in RD and JR1 cells 72 h post infection with pS-pre-miR-101 or control pS- retrovirus. Total α-tubulin and GAPDH were used as loading controls. Representative of three independent experiments. **d** Flow cytometry analysis after propidium iodide (PI) staining 72 h post infection with pS-pre-miR-101 or control pS- retrovirus on RD and JR1 cells was performed. Ten thousand events *per* sample were acquired. The histogram depicts the fold change of cells in the G1, S, and G2 phases after normalization to the percentage of GPF-positive cells for each sample. Results are means ± SD of two independent experiments

Next, we investigated *in vitro* whether miR-101 could regulate EZH2 expression in eRMS cells, as reported for other types of human cancers [12, 13]. We obtained an about 6-fold increase of miR-101 expression by infecting RD and JR1 cells with a GFP-coding retroviral vector expressing the pre-miR-101-2 form (pS-pre-miR-101) [19] (Fig. 3a and Additional file 2: Figure S2 for the efficiency of infection). Over-expression of miR-101 in eRMS cell lines induced a 30 % down-regulation of EZH2 mRNA and reduced protein levels compared to cells infected with an empty retrovirus (pS-) (Fig. 3b,c). Moreover, forced expression of miR-101 for 72 h resulted in the up-regulation of protein levels of the cyclin-dependent kinase inhibitor p21^Cip1^ (Fig. 3c). Therefore, we sought to evaluate whether miR-101 ectopic over-expression might affect eRMS cell proliferation. As reported in Fig. 3d, miR-101 over-expression determined a cell cycle G1/S blockade in RD cells whose percentage in G1 phase increased by 10 ± 3 % while in S and G2 phases decreased by 13 ± 2 and 2 ± 0.8 %, respectively, compared to pS- cells (Fig. 3d). These results are similar to those previously published by our group on RD cells after EZH2 silencing [11]. Interestingly, in JR1 cells in which miR-101 has been over-expressed, we noticed a cell cycle blockade in G2 phase (11.2 ± 1.8 % of increase), compared to pS- cells (Fig. 3d). Of note, the transcript levels of the oncogene N-Myc, a recognized miR-101 target gene in cancer [20] and involved in the aggressiveness of RMS [21], were markedly reduced in miR-101-over-expressing RD cells (Additional file 3: Figure S3A), confirming a targeted effect of miR-101 forced expression also in our setting. Altogether, these data suggest a reciprocal regulation between EZH2 and miR-101 in eRMS cells and indicate that miR-101 induction hampers their proliferative potential.

### Over-expression of miR-101 restrains the migration of embryonal RMS cells *in vitro*

**Fig. 4:**
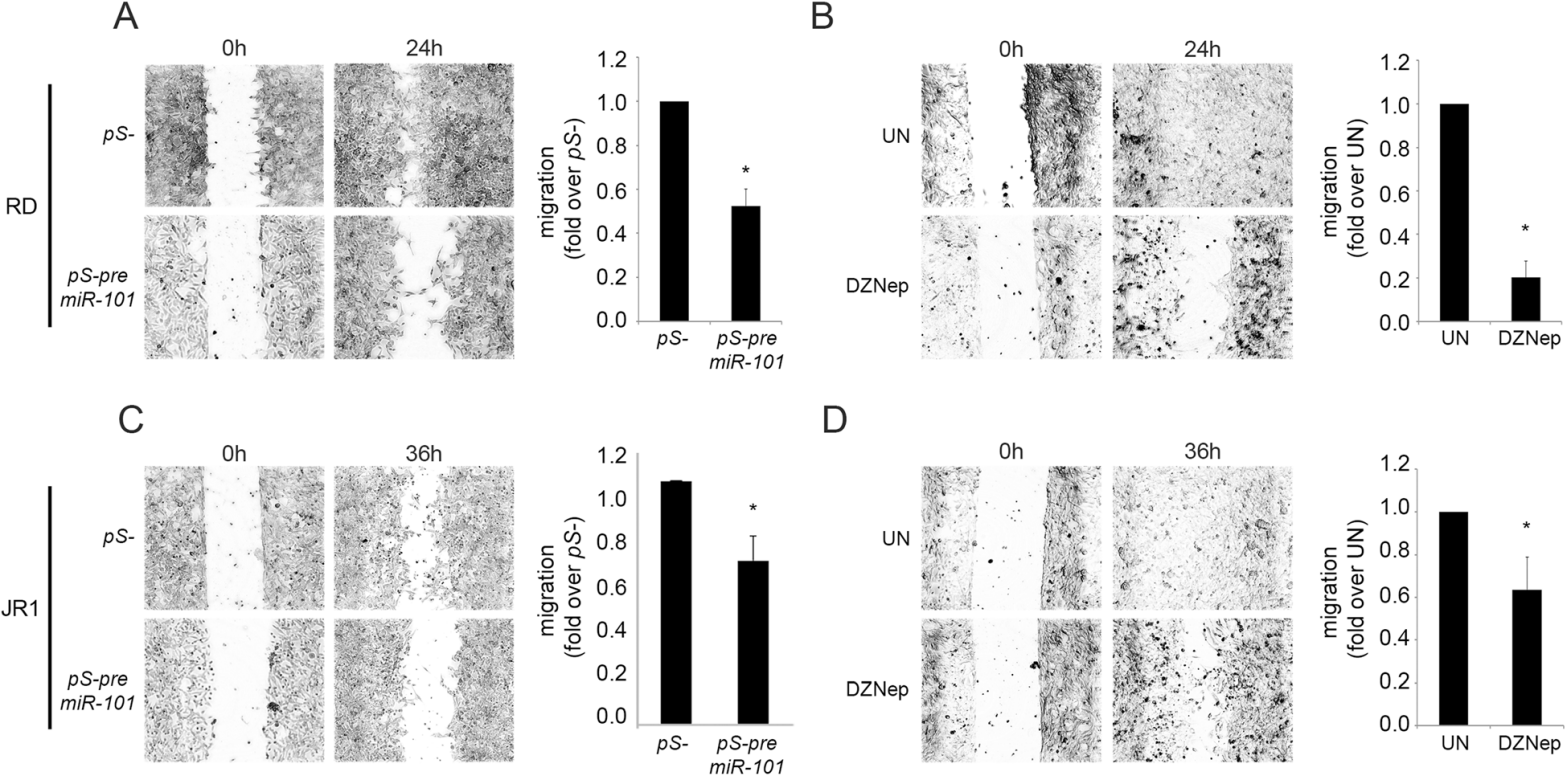
MiR-101 forced expression as well as EZH2 pharmacological inhibition reduces eRMS cell migration. RD (**a**) and JR1 (**c**) cells were infected with pS-pre-miR-101 or control pS- retrovirus. Twenty-four hours post-infection cells were seeded on inserts and lived to reach the confluence for 48 h, when the inserts were removed. Cells were imaged at 0 and 24 h or 36 h after the insert removal. RD (**b**) and JR1 (**d**) were treated with DZNep (5 μM) or vehicle (i.e., water, referred as untreated condition: UN) for 72 h and then inserts were removed and cells were imaged as in (**a**) and (**b**). Representative phase contrast microscopy images of the migration assays at 0 and 24 h or 36 h after gap creation were shown. *Dashed lines* indicate the boundary of the edges of the wound at 0 and 24 h or 36 h. The histograms depict the measurements of the total area between the wound edges of the scratch from at least five random fields per scratch from two separate experiments, expressed as fold change over control pS- (**a** and **c**, 1 arbitrary unit) or untreated (UN) (**b** and **d**, 1 arbitrary unit) samples. *Columns*, means; *bars*, SD. *P < 0.05 (Student’s *t*-test)

The miR-101 tumor-suppressive activities have been also related to its ability to negatively modulate tumor cell migration [22–24]. Therefore, we decide to evaluate the effects of miR-101 over-expression on the migratory potential of eRMS cells in a wound healing assay. The 24-h migration rate of pS-pre-miR-101-infected cells was reduced of about 40 and 30 % for RD and JR1 cells, respectively, compared to pS- cells (Fig. 4a,c). To determine whether EZH2 might be involved in their migratory capability, eRMS cells were treated with DZNep and the migration rate measured. As observed for miR-101 over-expression, EZH2 pharmacological inhibition reduced eRMS cell migration (70 and 35 % reduction for RD and JR1 cells, respectively) (Fig. 4b,d). In agreement with the effects of DZNep on miR-101 expression reported in Fig. 2b, DZNep-treated RD cells showed a down-regulation of the miR-101 target gene N-Myc (Additional file 3: Figure S3B). Altogether, these findings suggest that miR-101 and EZH2 regulate the migration of eRMS cells in an opposite manner.

### Over-expression of miR-101 reduces embryonal RMS cell tumorigenic potential

**Fig. 5:**
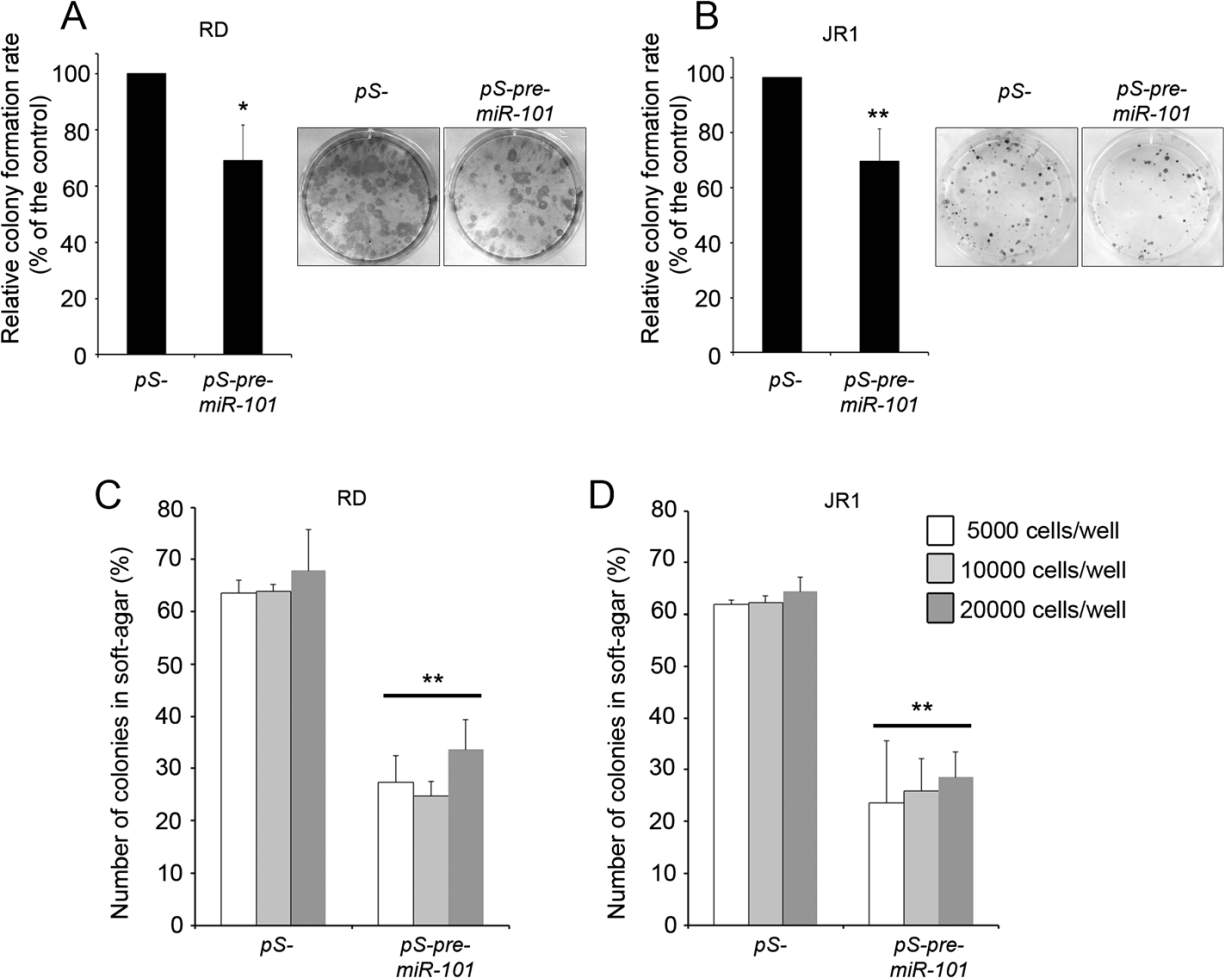
MiR-101 over-expression reduces colony formation and anchorage-independent growth capabilities in eRMS cells. RD (**a**) and JR1 (**b**) cells were infected with pS-pre-miR-101 or control pS- retrovirus and, 72 h later, seeded to examine their clonogenic capability 2 weeks post seeding (see “Methods” section). Histograms depict the number of colonies per plate from four independent experiments. Representative pictures of stained colonies were shown on the *right*. RD (**c**) and JR1 (**d**) cells, infected as in (**a**) and (**b**), were seeded on soft agar for an anchorage-independent growth assay. Colonies were visible. Histograms depict the number of colonies per plate after 4 weeks of incubation, calculated as means ± SD from four independent experiments. *Columns*, means; *bars*, SD. *P < 0.05, **P < 0.01 (Student’s *t*-test)

As a negative regulator of proliferation and migration, miR-101 is predicted to reduce tumorigenicity of eRMS cells. To test whether miR-101 restoration might restrain the clonogenic ability of eRMS cells, we performed colony formation assays with RD and JR1 cells over-expressing miR-101. As reported in Fig. 5a,b, miR-101 over-expression reduced of 30 % of the ability to form colonies in both RD and JR1 cells. Then, we evaluated the capability of eRMS cell lines over-expressing miR-101 to grow as colonies in soft agar in an anchorage-independent manner, indicative of malignant transformation and considered an *in vitro* surrogate of the *in vivo* tumorigenicity testing. As shown in Fig. 5c, d, miR-101 over-expression reduced the formation of colonies in soft agar of about 50 % in both RD and JR1 cells. Consistently, miR-101 over-expressing RD18 cells showed 50 % EZH2 down-regulation associated to cell cycle slow-down (5.4 ± 0.6 % increase of cells in the G1 phase and 14 ± 2 and 3.4 ± 0.6 % decrease in S and G2 phases, respectively) and a more modest but significant reduction of colony formation of about 20 and 15 % on either in culture dishes or soft agar (Additional file 4: Figure S4). Taken together, these results indicate that restoration of miR-101 in eRMS exerts an antitumor effect *in vitro*.

### MiR-101 expression is directly repressed by EZH2 in embryonal RMS

**Fig. 6:**
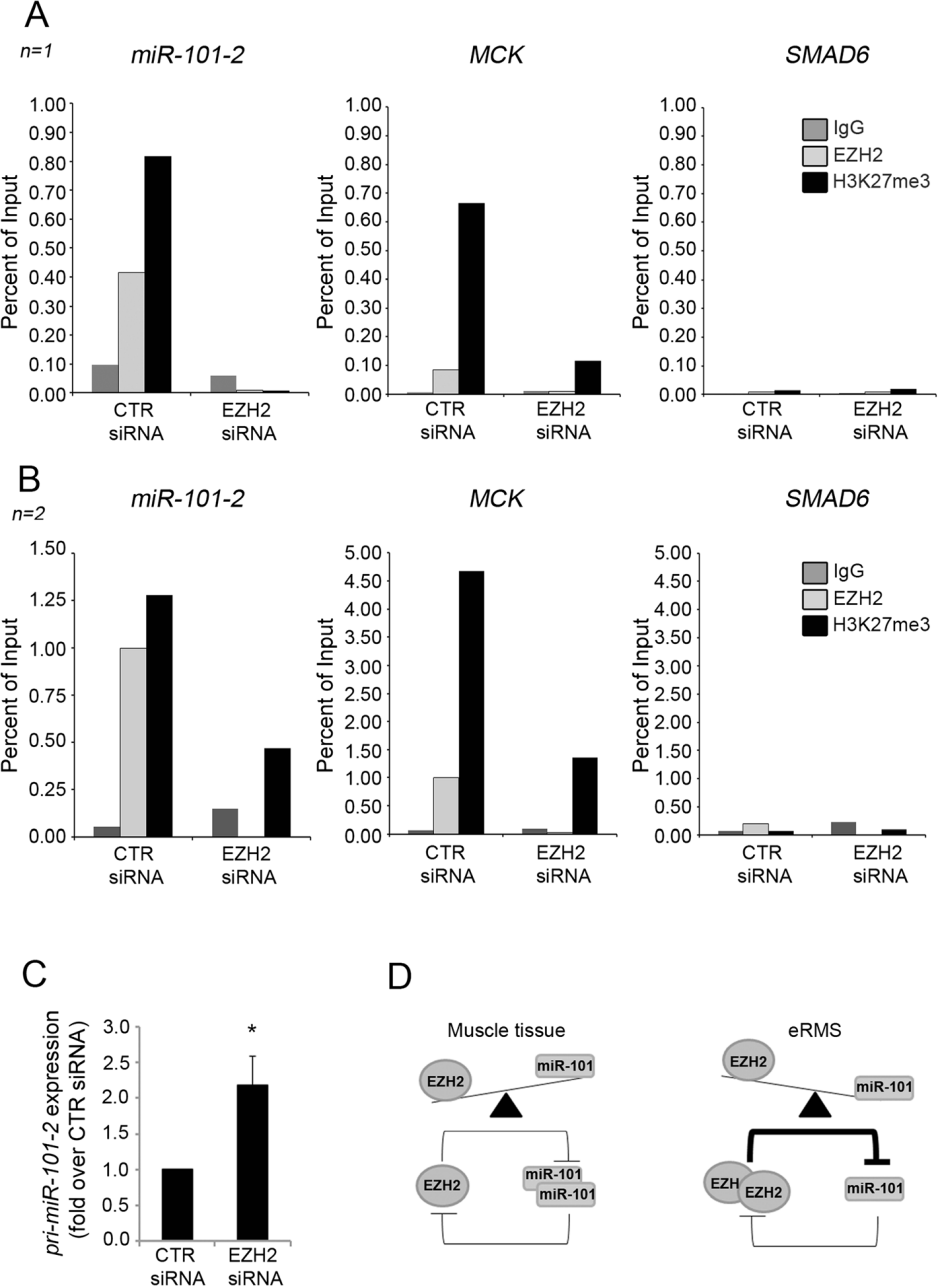
MiR-101 is directly targeted by EZH2 in RD cells. **a**, **b** Two independent ChIP assays on RD cells 72 h after EZH2 or CTR siRNA transfection showing the recruitment of EZH2 and histone H3 trimethylation on Lys27 (H3K27me3) levels on *miR-101-2* promoter region and *MCK* regulatory regions. *SMAD6* was the negative control gene. Rabbit IgG was used as a negative immunoprecipitation control. Histograms represent the percent of immunoprecipitated material relative to input DNA of the two independent experiments. **c** mRNA levels (RT-qPCR) of *pri*-*miR-101-2* in RD cells 72 h after EZH2 siRNA treatment were normalized to GAPDH levels and expressed as fold increase over CTR siRNA. **d** Proposed model depicting the interplay between EZH2 and miR-101 in both normal myogenic differentiation (*left*) and eRMS (*right*). In muscle cells, when myogenesis is triggered, miR-101 is upregulated due to the lowering of EZH2 expression. Then, miR-101 directly inhibits EZH2 expression thus enforcing its own expression, driving late skeletal muscle differentiation. In eRMS, this circuit is dysregulated due to EZH2 over-expression, which leads to miR-101 down-regulation, thus maintaining the cells in an undifferentiated and proliferative state

Since EZH2 down-regulation by either gene silencing or pharmacological inhibition induces miR-101 up-regulation (Fig. 2), we asked whether EZH2 might directly repress the expression of miR-101 in eRMS. To test this hypothesis, we performed chromatin immunoprecipitation (ChIP) experiments upon EZH2 silencing in RD and JR1 cells testing the occupancy of EZH2 on the promoter of *miR-101-2* that codifies for the miR-101 precursor *pri-miR-101-2* from which we derived the *pre-miR-101-2* vector used for over-expression experiments [25, 26]. As shown in two independent experiments reported in Fig. 6a, b, the promoter of the *miR-101-2* was occupied by EZH2 in RD cells and upon EZH2 siRNA, in accordance with EZH2 binding reduction to the promoter, also the level of H3K27me3 resulted strikingly reduced. Similar results were obtained in JR1 cells (Additional file 5: Figure S5). EZH2 silencing induced the up-regulation of the *pri-miR-101-2* in RD (Fig. 6c) and in JR1 (Additional file 5: Figure S5B) cells further corroborating the de-repression effect of EZH2 depletion.

## Discussion

In this study, we report, for the first time, that the microRNA miR-101 is down-regulated in the most recurrent variant of pediatric soft tissue sarcoma, i.e., the embryonal rhabdomyosarcoma (eRMS), showing an inverse pattern of expression with the histone methyltransferase EZH2. This latter is a miR-101 target gene [12] and behaves as an oncogene in eRMS [11, 27]. Moreover, we unveil a new functional connection between miR-101 and EZH2 in this tumor context. We demonstrate that knockdown of EZH2 by RNA silencing is sufficient to induce the up-regulation of the endogenous levels of miR-101 in eRMS cells, thus suggesting that EZH2 might repress miR-101 in this tumor type, as reported for other cancers [12, 25]. This evidence was confirmed by the induction of miR-101 expression also in tumor cells in which EZH2 was down-regulated through the treatment with DZNep, a compound which works inducing EZH2 degradation and already validated as an inhibitor of EZH2 by our group on the same context [11]. The concomitant induction in EZH2-depleted eRMS cells of the myogenic microRNAs miR-214 and miR-29b, which have been previously involved in negative feedback loops with EZH2 in myoblasts and RMS cells [3, 6, 8], confirms the disruption of EZH2-dependent tumorigenic pathways. Interestingly, while the up-regulation of miR-29b and miR-214 was comparable among the three cell lines, the de-repression of miR-101 appeared more modest in JR1 compared to RD and RD18 cells, suggesting a context-dependent response. Then, we show that retroviral-mediated forced expression of a precursor of mature miR-101, which is known to target EZH2 (pre-miR-101-2), in eRMS cells results in the down-regulation of both mRNA and protein levels of EZH2. MiR-101 has been reported to exert tumor suppressor functions in several human cancers by modulating EZH2 expression [12, 13, 28–31]. Therefore, on one hand, these results demonstrate that miR-101 is able to regulate EZH2 levels also in the eRMS tumor cell context and, on the other hand, they shed light on the molecular mechanisms by which EZH2 could be up-regulated in eRMS. This scenario might suggest that, in these tumor cells, EZH2 must be depleted in order to allow miR-101 increase.

Coherently with the evidence that in eRMS cells (i) EZH2 depletion inhibits proliferation (our previous report [11]) and (ii) forced induction of miR-101 down-regulates EZH2 (the present manuscript), we noticed a reduction in the growth rate of miR-101 over-expressing eRMS cells. The antiproliferative effect of miR-101 forced expression in these cells might be related to the increase in p21^Cip1^ levels, which can regulate both G1 or G2 cell cycle blockade, the same effect that we previously observed upon EZH2 silencing [11]. However, this aspect needs to be confirmed in future studies. Based on our observations, it can be hypothesized that low levels of miR-101 in eRMS contribute to the up-regulation of EZH2, which sustains tumor cell proliferation. Consistently with this hypothesis, EZH2 genetic of pharmacologic inhibition induces the blockade of eRMS cell proliferation and the appearance of a muscle-like phenotype [11].

This finding is in line with the evidence that (i) miR-101 expression increases in human SKMC induced to differentiate, i.e., cell cycle arrested, which confirms the recent observations obtained through microRNA profiling [18], and, in turn, (ii) EZH2 expression decreases in the same context, as previously reported by us and others [5, 6, 32].

However and interestingly, even if miR-101 increases in RD cells depleted of EZH2, its forced induction is unable to promote terminal differentiation *in vitro* and myosin heavy chain (MHC)-positive myotube-like fiber formation (data not shown). Nevertheless, the myogenic role of miR-101 has not yet been defined. As a matter of fact, although miR-101 was barely detectable in murine myoblasts in proliferation, its expression was not modulated during myogenic cell differentiation [6]. Clearer is the role of miR-101 in inhibiting tumor cell migration [23, 33]. Consistently with its tumor suppressor properties, when over-expressed miR-101 significantly reduced eRMS cell motility *in vitro*. Similar results were obtained by pharmacologically down-regulating EZH2, once again confirming the opposite functional roles of EZH2 and miR-101 in these tumor cells. Our results also unveil an inhibitory effect of miR-101 on the tumorigenic potential of eRMS cells by blocking both the clonogenic capability and the anchorage-independent features typical of malignant cells. Finally, the evidence that EZH2 binds the miR-101 gene promoter highlighted a direct effect of the oncogene on miR-101 expression further supporting a feedback loop involving the two molecular players. In summary, our findings indicate that EZH2 represses miR-101 expression and that, in turn, miR-101 can restrain EZH2 expression in eRMS (Fig. 6d).

## Conclusions

Results presented here now unveil miR-101 low expression as a new epigenetic dysregulation in eRMS and highlight its tumor suppressor role in this tumor type. We show that miR-101 is directly repressed by EZH2, a key player whose targeting has been suggested as a powerful epigenetic therapy to halt eRMS tumorigenicity. Although the precise role of miR-101 in myogenesis still requires in-depth investigation, results presented here indicate that a fine tuning regulation of the levels of EZH2 and miR-101 is critical for defying miR-101/EZH2 functional balance in eRMS, thus reinforcing the concept that epigenetic dysregulation is a key event in the pathogenesis of this tumor.

## Methods

### Cell lines

RD (embryonal RMS, eRMS) cell lines were obtained from American Type Culture Collection (Rockville, MD). RD18, JR1, and RUCH2 (all eRMS) cell lines were a gift of C. Ponzetto, G. Grosveld, and J. Roma, respectively. Normal human skeletal muscle cells (SkMC; myoblasts) were obtained from PromoCell (Promocell GmbH, Heidelberg, Germany).

### Cell line culture

RD, RUCH2, and RD18 cells were cultured in DMEM high glucose while JR1 cells were cultured in RPMI 1640 (both from Invitrogen Corp., Carlsbad, CA, USA). All RMS cell lines were cultured in medium supplemented with 10 % FCS, 1 % glutamine, and 1 % penicillin-streptomycin at 37 °C in a humidified atmosphere of 5 % CO_2_/95 % air. Human myoblasts, SkMC (C-12530 PromoCell GmbH, Heidelberg, Germany), were maintained in proliferating condition in PromoCell Cell Growth Medium (GM) supplemented with growth factors (C-23060 and C23160, PromoCell GmbH, Heidelberg, Germany). A human skeletal muscle differentiation model was obtained treating SkMC myoblasts for 14 days with a differentiating medium (DM) with appropriate supplements (C-23161 and C-39366, PromoCell GmbH, Heidelberg, Germany). Several aliquots of the first culture for each RMS cell line were stored in liquid nitrogen at −80 °C for subsequent assays. Each aliquot was passaged for a maximum of 5 months. ATCC genomics core utilizes scientific knowledge and technical expertise to design and perform numerous authentication and confirmatory assays (such as DNA barcoding and species identification, quantitative gene expression and transcriptome analyses) for ATCC collections (see www.lgcstandards-atcc.org). The DSMZ authenticates all human cell lines prior to accession by DNA typing, while the species-of-origin of animal cell lines are confirmed by PCR analysis (“speciation”). Independent evidence of authenticity is also provided by cytogenetic and immunophenotypic tests of characterization which are particularly informative among human tumor cell lines which form the bulk of the collection (see www.dsmz.de). Five different batches of SkMC were obtained, each from a different healthy donor, and immediately cultured and assayed in specific experiments as reported. The cell factory departments tested cells for cell morphology, adherence rate, and cell viability; immunohistochemical tests for cell-type-specific markers are carried out for each lot and, furthermore, the capacity to differentiate into multinucleated syncytia is routinely checked for each lot (see www.promocell.com).

### RMS primary tissues

RMS and control tissues were obtained from the Clinical Oncohematology Division, Ospedale Pediatrico Bambino Gesù in Rome, Italy, and Oncohematology Department, Vall d’Hebron Hospital in Barcelona, Spain, after approval of the respective ethical committees (EC of Ospedale Pediatrico Bambino Gesù, Rome; CEIC of Vall d’Hebron Hospital). Clinicopathological characteristics of the cohort are reported in Additional file 6: Table S1. We confirm that written informed consent from the donor or the next of kin was obtained for use of these samples in research.

### Real-time RT-quantitative PCR

Total RNA was extracted using TRIzol (Invitrogen, Carlsbad, CA, USA) according to the manufacturer’s protocol and inspected by agarose gel electrophoresis. Reverse transcription was performed using the Improm-II Reverse Transcription System (Promega, Madison, WI, USA). The expression levels were measured by real-time RT-qPCR for the relative quantification of the gene expression as described [9]. TaqMan gene assay (Applied Biosystems, Life Technologies, Carlsbad, CA, USA) for EZH2 (Hs01016789_m1), N-Myc (Hs00232074_m1), and pri-miR-101-2 (Hs03303387_pri) were used. The samples were normalized according to the glyceraldehyde-3-phosphate dehydrogenase (GAPDH) mRNA (Hs99999905_m1) levels.

Reverse transcription for miRNAs was performed using the TaqMan MicroRNA Reverse Transcription Kit with specific miRNA primers (Applied Biosystems). TaqMan microRNA assays (Applied Biosystems) were used for relative quantification of the mature miR-101 (hsa-miR-101; 002253), miR-29b (hsa-miR-29b; 0000413), and miR-214 (hsa-miR-214; 002293) expression levels, as described [9]. snoU6 snRNA (001093) was used for normalization. An Applied Biosystems 7900HT Fast Real-Time PCR System (Applied Biosystems) was used for the measurements. The expression fold change was calculated by the 2^-ΔΔCt^ method for each of the reference genes [34]. At least two independent amplifications were performed for each probe, with triplicate samples.

### Western blotting

Western blotting was performed on whole-cell lysates as previously described [35, 36]. Total protein extraction was performed by homogenizing cells in RIPA lysis buffer (50 mM Tris pH 7.5, 150 mM NaCl, 1 % Triton X-100, 1 mM EDTA, 1 % sodium deoxycholate and phosphatases 1 % cocktail protease inhibitors, 0.5 mM sodium orthovanadate). Lysates were sonicated and incubated on ice for 30 min and centrifugated at 12,000 g for 20 min at 4 °C. Supernatants were then quantified with BCA Protein Assay Kit (Pierce, Life Technologies) according to the manufacturer’s protocol and then boiled in reducing SDS sample buffer (200 mM Tris–HCl [pH 6.8], 40 % glycerol, 20 % β-mercaptoethanol, 4 % sodium dodecyl sulfate, and bromophenol blue); and 30 μg of protein lysate per lane was run through 7 and 12 % SDS-PAGE gels, and then transferred to Hybond ECL membranes (Amersham, GE HEALTHCARE BioScience Corporate Piscataway, NJ, USA). Membranes were blocked for 1 h in 5 % non-fat dried milk in Tris-buffered saline (TBS) and incubated overnight with the appropriate primary antibody at 4 °C. Membranes were then washed in TBS and incubated with the appropriate secondary antibody. Both primary and secondary antibodies were diluted in 5 % non-fat dried milk in TBS. Detection was performed by ECL Western Blotting Detection Reagents or by ECL Plus Western Blotting Detection Reagents (Amersham, GE HEALTHCARE BioScience Corporate Piscataway, NJ, USA). Antibodies against EZH2 (612666; Transduction Laboratories TM, BD, Franklin Lakes, NJ), p21 (C-19) (sc-397; Santa Cruz Biotechnology Inc., Santa Cruz, CA, USA), α-tubulin (NB 100–92249, Novus Biologicals), and GAPDH (D16H11; Cell Signaling Technology Inc., Beverly, MA, USA) were used. All secondary antibodies were obtained from Santa Cruz Biotechnology (Santa Cruz Biotechnology, Inc., Santa Cruz, CA, USA). All the antibodies were used in accordance with the manufacturer’s instructions. Images of radiograms were acquired through the HP Precision ScanJet 5300 C Scanner (Hewlett-Packard, Palo Alto, CA, USA).

### Transient RNA interference transfection and pharmacological treatments

Cells were seeded in 6-well/plates (150,000 cells/well) and grown up to 30 % confluence. After 24 h, cells were transfected with ON-TARGETplus SMART pool siRNA against EZH2 (L-004218-00) or non-targeting siRNA (control; D-001206-13) (both from Dharmacon, Thermo Fisher Scientific, Lafayette, CO, USA) or with a siRNA targeting the 5′-UTR of EZH2 mRNA with the following sequence 5′-CGGTGGGACTCAGAAGGCA-3′ and non-targeting siRNA as control (5′-UGGUUUACAUGUCGACUAA-3′) (both from Sigma, St Louis, MO, USA) [32] at 100 nM final concentration each round using Oligofectamine (Invitrogen, Carlsbad, CA), according to manufacturer’s recommendations. After 24 h, cells were transfected again and siRNA effectiveness was validated by Western blotting and RT-qPCR 48 h after the first silencing. For pharmacological treatments, cells were treated with either 5 μM deazaneplanocin A (DZNep) or water as vehicle for 48 h or 72 h.

### Virus production and cell infections

pSuper.retro vector expressing the endogenous human miR-101-2 precursor (pS-pre-miR-101) and its negative control (pS-, empty) have been already described [17, 19]. These vectors were transfected into Phi-NX (“Phoenix”) packaging cell line to produce ecotropic retroviral supernatants. Phoenix cells were cultured in Dulbecco’s modified Eagle’s medium (DMEM) supplemented with 10 % FCS. The day before transfection, Phoenix cells were seeded in 10-cm dishes (5 × 10^6^ cells/dish) in order to reach 85–90 % confluence at the time of transfection. Cells were transfected with 10 μg of viral vector DNA using Lipofectamine 2000 transfection reagent (Invitrogen, Carlsbad, CA, USA) according to the manufacturer’s instructions. After 6 h of incubation at 37 °C, transfection medium was replaced with 7 ml of complete medium containing 10 % FCS. At 48 h after transfection, culture medium was filtered through a 0.45-mm filter and the viral supernatant was used for RD, JR1, and RD18 cell infection after addition of 8 mg/ml of polybrene (Sigma, St Louis, MO, USA). After infection, RD, JR1, and RD18 cells were incubated at 37 °C in 5 % CO_2_. After 8 h of incubation, the medium was changed with new viral supernatants and incubated overnight. Then, the medium was changed with a fresh medium and cells were allowed to recover for 24 h at 37 °C in 5 % CO_2_. Infection efficiency was examined under a fluorescence microscope (not shown) and determined by flow cytometry for the expression of the green fluorescent protein (GFP). MiR-101 expression levels in RD, JR1, and RD18 cells infected with the control (−pS) and miR-101 expressing vector (pS-pre-miR-101) was analyzed by real-time polymerase chain reaction (RT-qPCR).

### Cell cycle assays

After two rounds of infection with the control (−pS) and miR-101 expressing vector (pS-pre-miR-101), RD, JR1, and RD18 cells were analyzed by flow cytometry as reported [37]. Briefly, cells were harvested by trypsinization 72 h after infection, washed in ice-cold PBS, fixed in 50 % PBS and 50 % acetone/methanol (1:4 *v*/*v*) for at least 1 h, and, after removing alcoholic fixative, stained in the dark with a solution containing 50 μg/ml propidium iodide (PI) and 50 μg/ml RNase (Sigma Chemical Co., St Louis, MO, USA) for 30 min at room temperature. The stained cells were analyzed for cell cycle by fluorescence-activated cell sorting using a FACSCantoII equipped with a FACSDiva 6.1 CellQuest™ software (Becton Dickinson Instrument, San Josè, CA, USA). The percentage of cells in G0/G1, S, and G2/M phases was expressed as relative change compared to pS-infected cells, and normalized to the percentage of GPF-positive cells as measured by flow cytometry.

### Cell wound healing assay

Wound healing assay was performed with the Ibidi Culture-Insert (Ibidi®) as manufacturer’s instruction. Briefly, cell suspensions of RD and JR1 cells infected with pS-pre-miR-101 and pS- or treated with DZNep/Vehicle for 72 h were prepared (3–4 × 10^5^ cells/ml) and 70 μl were applied into each well. Cells were incubated at 37 °C and 5 % CO_2_ for 24 h. After appropriate cell attachment, culture inserts were gently removed, fresh medium was added, and images were captured immediately (day 0) and 24 and 36 h later with a Leica DMi8 Inverted Microscope. Cell migration was quantitatively assessed measuring the entire area of the scratches by ImageJ software (Wayne Rasband, NIH, Bethesda, MD, USA; http://rsb.info.nih.gov/ij/). The results were obtained from measurements of the total area of the scratch between the wound edges per scratch from two separate experiments for each cell line, expressed as fold change over either control ones.

### Colony formation assay

After 72 h of infection with retroviral pS-pre-miR-101 and pS-, RD, JR1, and RD18 cells were assayed for the clonogenic survival. A total of 5 × 10^2^ or 10 × 10^2^ cells were seeded in 6 multi-well plates with 2 mL of DMEM (10 % FBS). Medium was refreshed every 2 days, and after 14 days, cells were fixed and stained with Diff-Quik® (Medion Diagnostic AG 460.053) as manufacturer’s instruction. Colonies containing >50 cells were counted. Triplicate assays were carried out in four independent experiments.

### Soft agar colony formation assay

After 72 h of infection with retroviral pS-pre-miR-101 and pS-, RD, JR1, and RD18 cells were assayed for their capacity to form colonies in soft agar. A total of 5 × 10^3^, 10 × 10^3^, or 20 × 10^3^ cells were suspended in DMEM (10 % FBS) containing 0.35 % agar (NuSieve GTG Agarose). Cells were seeded on a layer of 0.7 % agar in DMEM (10 % FBS) in 6 multi-well plates. Medium was refreshed every 2 days. On week 4, colonies were counted by microscopic inspection. Colony numbers were normalized by dividing the number of colonies by the number of total units (colonies + single cells). Triplicate assays were carried out in four independent experiments.

### Chromatin immunoprecipitation (ChIP)

ChIP assay was performed as previously described [11, 32] with minor modifications. Briefly, chromatin was cross-linked in 1 % formaldehyde for 15 min at room temperature and quenched by addition of glycine at 125 mM final concentration for 5 min at room temperature before being placed on ice. Cells were washed twice with ice-cold PBS containing 1 mM PMSF and 1X protease inhibitors, resuspended in ice-cold cell lysis buffer (10 mM Tris–HCl pH 8, 10 mM NaCl, 0.2 % NP-40, 1 mM PMSF, and 1X protease inhibitors), and incubated on ice for 30 min. After centrifugation at 4000 rpm for 5 min at 4 °C, nuclei were resuspended in ice-cold nuclear lysis buffer (50 mM TrisHCl pH 8.1; 10 mM EDTA; 1 % SDS, 1 mM PMSF, and 1X protease inhibitors) and left overnight at 4 °C on a rotating platform. Chromatin was then sonicated to an average fragment size of 200–300 bp using a Diagenode (water bath) and diluted ten times with IP dilution buffer (16.7 mM Tris–HCl pH 8.1, 167 mM NaCl, 1.2 mM EDTA, 0.01 %SDS, 1.1 % Triton X-100, 1 mM PMSF, and 1X protease inhibitors). Diluted chromatin was pre-cleared using protein G-agarose magnetic beads (Invitrogen) for 1 h at 4 °C and incubated with the corresponding antibodies overnight at 4 °C. The following antibodies were used: anti-trimethyl Lysine 27 histone H3 (Cell Signaling, #9733) and anti-EZH2 (Cell Signaling, #5246). Immunoprecipitated chromatin was recovered by incubation with protein G-agarose magnetic beads (Invitrogen, Carlsbad, CA, USA) for 2 h at 4 °C. Beads were washed twice with low-salt washing buffer (20 mM Tris–HCl pH 8, 2 mM EDTA, 1 % Triton X-100, 0.1 % SDS, 150 mM NaCl), twice with high-salt washing buffer (20 mM Tris–HCl pH 8, 2 mM EDTA, 1 % Triton X-100, 0.1 % SDS, 500 mM NaCl), and twice with TE before incubating them with elution buffer (10 mM Tris–HCl pH 8, 1 mM EDTA, 1 % SDS). Cross-linking was then reverted overnight at 65 °C and samples were treated with proteinase K for 2 h at 42 °C. The DNA was finally purified by phenol: chloroform extraction in the presence of 0.4 M LiCl and ethanol precipitated. Purified DNA was resuspended in 50 μl of water. Real-time PCR was performed on input samples and equivalent amounts of immunoprecipitated material with the SYBR Green Master Mix (Applied Biosystems, Life Technologies, Carlsbad, CA, USA). Primer sequences are available on request.

### Statistical analysis

The data were presented as the means ± SD. Comparisons were made between the means from at least two independent experiments repeated in triplicate. The statistical differences were analyzed using Student’s *t*-test. *P* values < 0.05 were considered statistically significant.

## Additional files

Additional file 1: Figure S1. EZH2 levels in eRMS cells after EZH2 down-regulation. Western blot showing the reduction of EZH2 levels in RD cells after 48 h of transfection with EZH2 pool siRNA(*) or a non-targeting control (CTR) siRNA (A), RD, JR1, and RD18 after 48 h of transfection with EZH2 5′UTR EZH2 siRNA or a non-targeting control (CTR) siRNA (B), or DZNep treatment (5 μM) or vehicle (i.e., water, referred as untreated condition: UN) (C). Total α-tubulin or GAPDH were used as loading controls. Representative of three independent experiments. Additional file 2: Figure S2. Infection efficiency in eRMS cells. Representative cytofluorometric plots show the level of GFP fluorescence in RD, JR1, and RD18 cells infected with pS-pre-miR-101 or control pS- retrovirus for 72 h, and the percentage of GFP positivity is reported inside the plots within the *right upper quadrant* Q2 and in the tables on the *right*. Additional file 3: Figure S3. RT-qPCR analysis of N-Myc in RD cells after miR-101 over-expression or EZH2 inhibition. (A) RT-qPCR analysis of N-Myc in RD cells infected with pS-pre-miR-101 and control pS- retrovirus. Data were normalized to GAPDH levels and expressed as fold increase over control (pS-, 1 arbitrary unit). (B) RD cells were treated with DZNep (5 μM) or vehicle (i.e., water, referred as untreated condition: UN). Data were normalized to GAPDH levels and expressed as fold increase over UN (1 arbitrary unit). *Columns*, means; *bars*, SD. Results from three independent experiments are shown. **P* < 0.05 (Student’s *t*-test). Additional file 4: Figure S4. miR-101 over-expression reduces EZH2 levels, cell proliferation, anchorage-independent growth, and cell proliferation in RD18 cells. qRT-PCR analysis of mature miR-101 (A) and EZH2 (B) 72 h post infection with pS-pre-miR-101 or control pS- retrovirus. Data were normalized using snoU6 and GAPDH levels respectively and expressed as fold increase over control (pS-, 1 arbitrary unit). *Columns*, means; *bars*, SD. Results from three independent experiments are shown. **P* < 0.05 (Student’s *t*-test). (C) Western blot showing EZH2 levels 72 h post infection with pS-pre-miR-101 or control pS- retrovirus. GAPDH was used as a loading control. Representative of three independent experiments. (D) Flow cytometry analysis after propidium iodide (PI) staining 72 h post infection with pS-pre-miR-101 or control pS- retrovirus was performed. Ten thousand events *per* sample were acquired. The histogram depicts the fold change of cells in the G1, S, and G2 phases after normalization using percentage of GPF-positive cells for each sample. Results are means ± SD of two independent experiments. (E) Proliferation assay shows a growth reduction after 72 h of infection with pS-pre-miR-101 vs control pS- retrovirus. Colonies were visible after 2 weeks (see “Methods” section) of incubation and values represent the number of colonies per plate calculated as means ± SD from four independent experiments. Representative colony formation pictures were shown. *Columns*, means; *bars*, SD. **P* < 0.05 (Student’s *t*-test). (F) Cells infected with pS-pre-miR-101 and control pS- retrovirus were examined for anchorage-independent growth by soft agar assay. Colonies were visible after 4 weeks (see “Methods” section) of incubation. The histograms represent the number of colonies per plate as means ± SD from four independent experiments. *Columns*, means; *bars*, SD. **P* < 0.05 (Student’s *t*-test). Additional file 5: Figure S5. Signal-specific recruitment of EZH2 onto the miR-101-2 promoter in JR1 cells after EZH2 down-regulation. (A) ChIP assays on JR1 cells 72 h after EZH2 or CTR siRNA transfection showing the recruitment of EZH2 and the levels of histone H3 trimethylation on Lys27 (H3K27me3) on *miR-101-2*, *MCK*, and *SMAD6* (as negative control) regulatory regions. Normal rabbit IgG were used as negative control. *Graphs* represent the percent of immunoprecipitated material relative to input DNA. (B) mRNA levels (RT-qPCR) of *pri*-*miR-101-2* in JR1 cells 48 h after EZH2 siRNA treatment were normalized to GAPDH levels and expressed as fold increase over CTR siRNA. Additional file 6: Table S1. Clinical and histopathologic data of rhabdomyosarcoma tumor.

## Abbreviations

RMS: rhabdomyosarcoma
eRMS: embryonal rhabdomyosarcoma
SKMC: skeletal muscle cells
GM: growth factor-supplemented medium
EZH2: enhancer of zeste of homologue 2
PRC2: polycomb repressor complex 2
GAPDH: glyceraldehyde 3-phosphate dehydrogenase
MCK: muscle creatine kinase
SMAD6: small mother against decapentaplegic 6
siRNA: small interfering RNA
DZNep: S-adenosylhomocysteine hydrolase inhibitor 3-deazaneplanocin A
SD: standard deviation
RT-qPCR: quantitative real-time polymerase chain reaction
PI: propidium iodide
