# Supplementary material for: MicroRNA-101 is repressed by EZH2 and its restoration inhibits tumorigenic features in embryonal rhabdomyosarcoma
Source: Clin Epigenetics. 2015 Aug 6;7(1):82. doi: 10.1186/s13148-015-0107-z (PMC4527101; doi:10.1186/s13148-015-0107-z)
Supplement: Additional file 1: Figure S1. — EZH2 levels in eRMS cells after EZH2 down-regulation. Western blot showing the reduction of EZH2 levels in RD cells after 48 h of transfection with EZH2 pool siRNA(*) or a non-targeting control (CTR) siRNA (A), RD, JR1, and RD18 after 48 h of transfection with EZH2 5′UTR EZH2 siRNA or a non-targeting control (CTR) siRNA (B), or DZNep treatment (5 μM) or vehicle (i.e., water, referred as untreated condition: UN) (C). Total α-tubulin or GAPDH were used as loading controls. Representative of three independent experiments. [file 13148_2015_107_MOESM1_ESM.pdf]

Figure S1

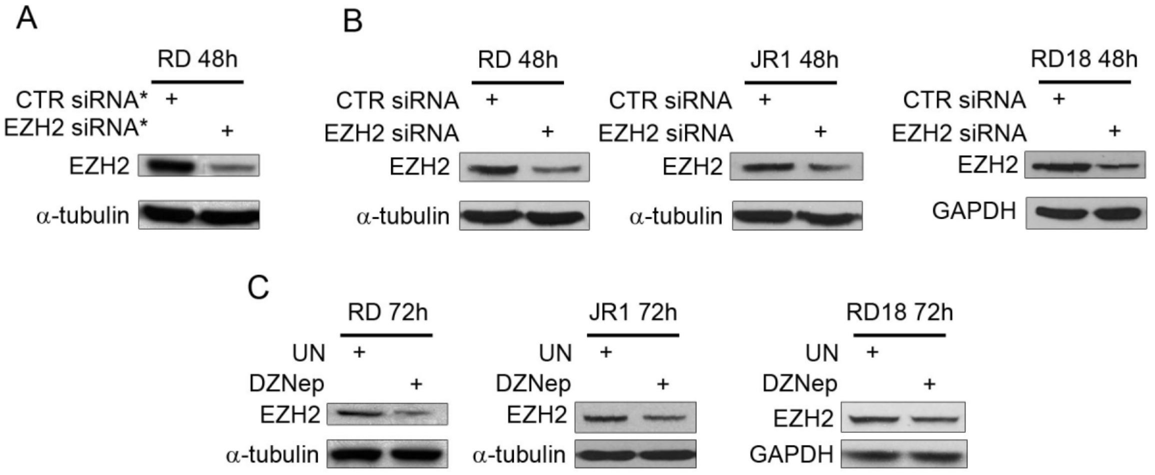

Additional file 1: Figure S1. EZH2 levels in eRMS cells after EZH2 down-regulation

Western blot showing the reduction of EZH2 levels in RD cells after 48h transfection with EZH2 pool siRNA(*) or a non-targeting control (CTR) siRNA (A), RD, JR1 and RD18 after 48h transfection with EZH2 5’UTR Ezh2 siRNA or a non-targeting control (CTR) siRNA (B), or DZNep treatment (5μM) or vehicle (i.e., water, referred as untreated condition: UN) (C). Total α-tubulin or GAPDH were used as loading controls. Representative of three independent experiments.
