# Supplementary material for: MicroRNA-101 is repressed by EZH2 and its restoration inhibits tumorigenic features in embryonal rhabdomyosarcoma
Source: Clin Epigenetics. 2015 Aug 6;7(1):82. doi: 10.1186/s13148-015-0107-z (PMC4527101; doi:10.1186/s13148-015-0107-z)
Supplement: Additional file 2: Figure S2. — Infection efficiency in eRMS cells. Representative cytofluorometric plots show the level of GFP fluorescence in RD, JR1, and RD18 cells infected with pS-pre-miR-101 or control pS- retrovirus for 72 h, and the percentage of GFP positivity is reported inside the plots within the right upper quadrant Q2 and in the tables on the right. [file 13148_2015_107_MOESM2_ESM.pdf]

Figure S2

RD

pS-

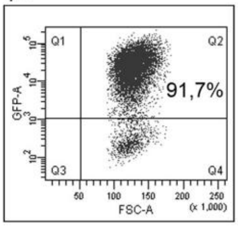

Population	#Events	%Parent
RD	20,021	81.8
Q1	0	0.0
Q2	18,355	91.7
Q3	0	0.0
Q4	1,666	8.3

pS-pre-miR-101

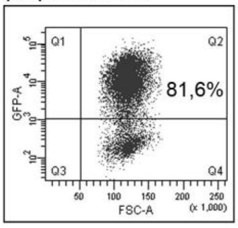

Population	#Events	%Parent
RD	20,440	70.6
Q1	0	0.0
Q2	16,674	81.6
Q3	0	0.0
Q4	3,766	18.4

JR1

pS-

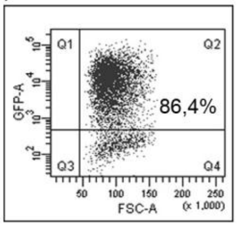

Population	#Events	%Parent
JR1	10,079	68.3
Q1	0	0.0
Q2	8,704	86.4
Q3	0	0.0
Q4	1,375	13.6

pS-pre-miR-101

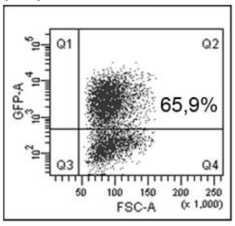

Population	#Events	%Parent
JR1	10,072	71.1
Q1	0	0.0
Q2	6,639	65.9
Q3	0	0.0
Q4	3,433	34.1

RD18

pS-

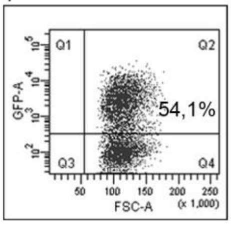

Population	#Events	%Parent
RD 18	10,000	79.0
Q1	32	0.3
Q2	6,848	54.1
Q3	488	3.9
Q4	5,288	41.8

pS-pre-miR-101

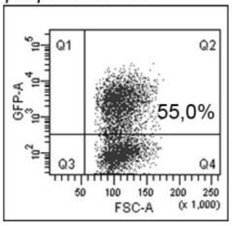

Population	#Events	%Parent
RD 18	10,000	81.6
Q1	0	0.0
Q2	5,499	55.0
Q3	0	0.0
Q4	4,501	45.0

Additional file 2: Figure S2. Infection efficiency in eRMS cells

Representative cytofluorometric plots show the level of GFP fluorescence in RD, JR1 and RD18 cells infected with pS-pre-miR-101 or control pS- retrovirus for 72h, and the percentage of GFP positivity is reported inside the plots within the right upper quadrant Q2 and in the tables on the right.
