# Supplementary material for: MicroRNA-101 is repressed by EZH2 and its restoration inhibits tumorigenic features in embryonal rhabdomyosarcoma
Source: Clin Epigenetics. 2015 Aug 6;7(1):82. doi: 10.1186/s13148-015-0107-z (PMC4527101; doi:10.1186/s13148-015-0107-z)
Supplement: Additional file 3: Figure S3. — RT-qPCR analysis of N-Myc in RD cells after miR-101 over-expression or EZH2 inhibition. (A) RT-qPCR analysis of N-Myc in RD cells infected with pS-pre-miR-101 and control pS- retrovirus. Data were normalized to GAPDH levels and expressed as fold increase over control (pS-, 1 arbitrary unit). (B) RD cells were treated with DZNep (5 μM) or vehicle (i.e., water, referred as untreated condition: UN). Data were normalized to GAPDH levels and expressed as fold increase over UN (1 arbitrary unit). Columns, means; bars, SD. Results from three independent experiments are shown. *P < 0.05 (Student’s t-test). [file 13148_2015_107_MOESM3_ESM.pdf]

Figure S3

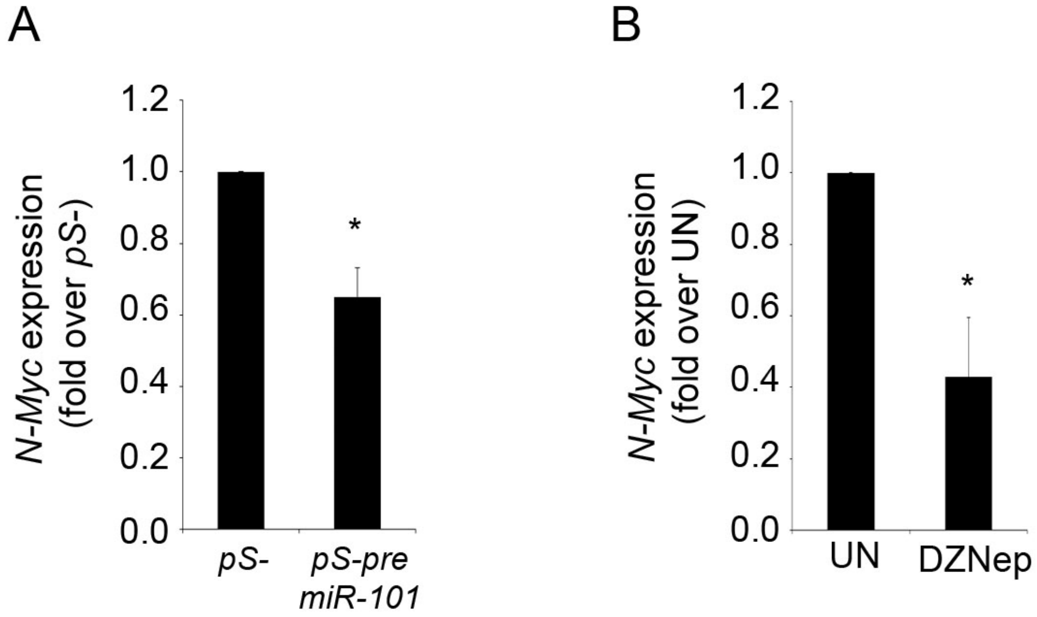

Additional file 3: Figure S3. qRT-PCR analysis of N-Myc in RD cells after miR-101 overexpression or EZH2 inhibition.

(A) qRT-PCR analysis of N-Myc in RD cells infected with pS-pre-miR-101 and control pS- retrovirus. Data were normalized to GAPDH levels and expressed as fold increase over control (pS-, 1 arbitrary unit). (B) RD cells were treated with DZNep (5μM) or Vehicle (i.e, water, referred as untreated condition: UN). Data were normalized to GAPDH levels and expressed as fold increase over UN (1 arbitrary unit). Columns, means; Bars, SD. Results from three independent experiments are shown. *$P < 0.05$ (Student’s t-test).
