# Supplementary material for: MicroRNA-101 is repressed by EZH2 and its restoration inhibits tumorigenic features in embryonal rhabdomyosarcoma
Source: Clin Epigenetics. 2015 Aug 6;7(1):82. doi: 10.1186/s13148-015-0107-z (PMC4527101; doi:10.1186/s13148-015-0107-z)
Supplement: Additional file 4: Figure S4. — miR-101 over-expression reduces EZH2 levels, cell proliferation, anchorage-independent growth, and cell proliferation in RD18 cells. qRT-PCR analysis of mature miR-101 (A) and EZH2 (B) 72 h post infection with pS-pre-miR-101 or control pS- retrovirus. Data were normalized using snoU6 and GAPDH levels respectively and expressed as fold increase over control (pS-, 1 arbitrary unit). Columns, means; bars, SD. Results from three independent experiments are shown. *P < 0.05 (Student’s t-test). (C) Western blot showing EZH2 levels 72 h post infection with pS-pre-miR-101 or control pS- retrovirus. GAPDH was used as a loading control. Representative of three independent experiments. (D) Flow cytometry analysis after propidium iodide (PI) staining 72 h post infection with pS-pre-miR-101 or control pS- retrovirus was performed. Ten thousand events per sample were acquired. The histogram depicts the fold change of cells in the G1, S, and G2 phases after normalization using percentage of GPF-positive cells for each sample. Results are means ± SD of two independent experiments. (E) Proliferation assay shows a growth reduction after 72 h of infection with pS-pre-miR-101 vs control pS- retrovirus. Colonies were visible after 2 weeks (see “Methods” section) of incubation and values represent the number of colonies per plate calculated as means ± SD from four independent experiments. Representative colony formation pictures were shown. Columns, means; bars, SD. *P < 0.05 (Student’s t-test). (F) Cells infected with pS-pre-miR-101 and control pS- retrovirus were examined for anchorage-independent growth by soft agar assay. Colonies were visible after 4 weeks (see “Methods” section) of incubation. The histograms represent the number of colonies per plate as means ± SD from four independent experiments. Columns, means; bars, SD. *P < 0.05 (Student’s t-test). [file 13148_2015_107_MOESM4_ESM.pdf]

Figure S4

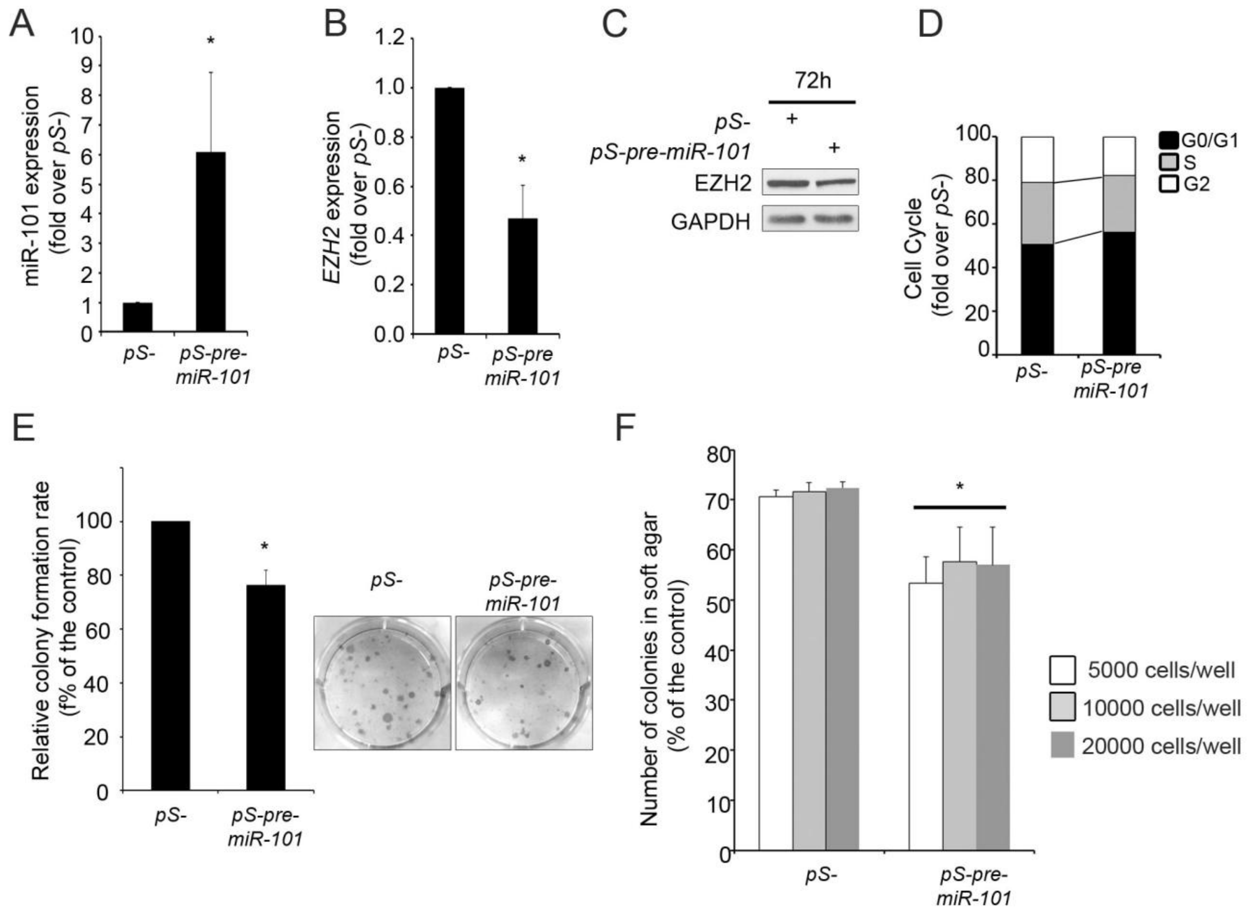

Figure S4. miR-101 overexpression reduces EZH2 levels, cell proliferation, anchorage independent growth and cell proliferation in RD18 cells.

qRT-PCR analysis of mature miR-101 (A) and EZH2 (B) 72h post infection with pS-pre-miR-101 or control pS- retrovirus. Data were normalized using snoU6 and GAPDH levels respectively and expressed as fold increase over control (pS-, 1 arbitrary unit). Columns, means; Bars, SD. Results from three independent experiments are shown. *$P < 0.05$ (Student's t-test). (C) Western blot showing EZH2 levels 72h post infection with pS-pre-miR-101 or control pS-retrovirus. GAPDH was used as loading control. Representative of three independent experiments. (D) Flow citometry analysis after Propidium Iodide (PI) staining 72h post infection with pS-pre-miR-101 or control pS-

retrovirus was performed. Ten thousand events *per* sample were acquired. The histogram depicts the fold change of cells in the G1, S and G2 phases after normalization using percentage of GPF-positive cells for each sample. Results are means ± SD of two independent experiments.

(E) Proliferation assay shows a growth reduction after 72h infection with pS-pre-miR-101 vs control pS- retrovirus. Colonies were visible after 2 weeks (see methods) of incubation and values represent the number of colonies per plate calculated as mean ±SD from four independent experiments. Representative colony formation pictures were shown. Columns, means; Bars, SD $*P < 0.05$ (Student's t-test). (F) Cells infected with pS-pre-miR-101 and control pS- retrovirus were examined for anchorage-independent growth by soft-agar assay. Colonies were visible after 4 weeks (see methods) of incubation. The histograms represent the number of colonies per plate calculated as mean ±SD from four independent experiments. Columns, means; Bars, SD, $*P < 0.05$ (Student's t-test).
