# Supplementary material for: MicroRNA-101 is repressed by EZH2 and its restoration inhibits tumorigenic features in embryonal rhabdomyosarcoma
Source: Clin Epigenetics. 2015 Aug 6;7(1):82. doi: 10.1186/s13148-015-0107-z (PMC4527101; doi:10.1186/s13148-015-0107-z)
Supplement: Additional file 5: Figure S5. — Signal-specific recruitment of EZH2 onto the miR-101-2 promoter in JR1 cells after EZH2 down-regulation. (A) ChIP assays on JR1 cells 72 h after EZH2 or CTR siRNA transfection showing the recruitment of EZH2 and the levels of histone H3 trimethylation on Lys27 (H3K27me3) on miR-101-2, MCK, and SMAD6 (as negative control) regulatory regions. Normal rabbit IgG were used as negative control. Graphs represent the percent of immunoprecipitated material relative to input DNA. (B) mRNA levels (RT-qPCR) of pri-miR-101-2 in JR1 cells 48 h after EZH2 siRNA treatment were normalized to GAPDH levels and expressed as fold increase over CTR siRNA. [file 13148_2015_107_MOESM5_ESM.pdf]

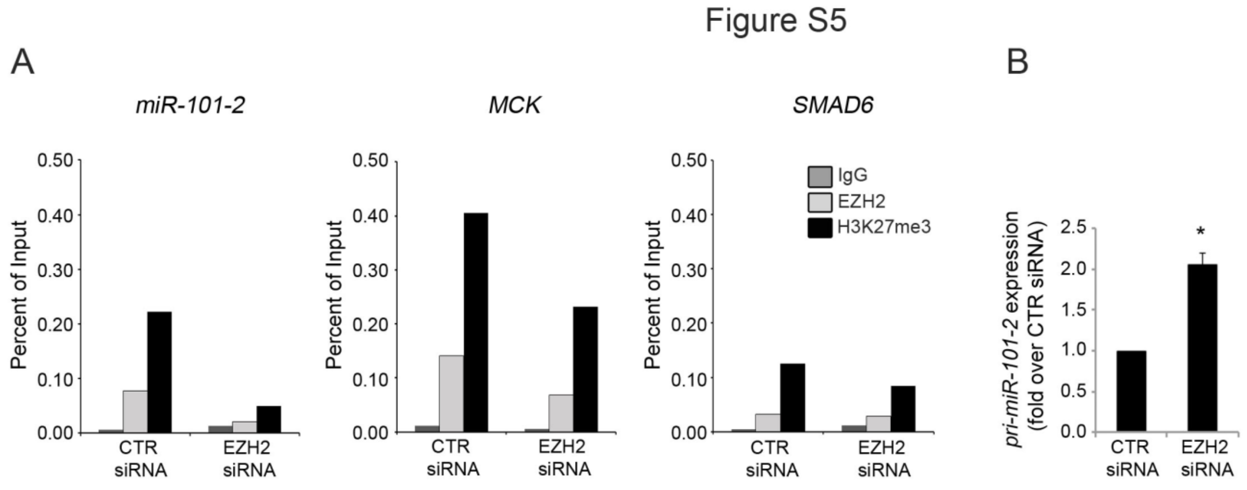

Figure S5. Signal-specific recruitment of EZH2 onto the miR-101-2 promoter in JR1 cells after EZH2 down-regulation

(A) ChIP assays on JR1 cells 72 h after EZH2 or CTR siRNA transfection showing the recruitment of EZH2 and the levels of histone H3 trimethylation on Lys27 (H3K27me3) on *miR-101-2, MCK* and *SMAD6* (as negative control) regulatory regions. Normal rabbit IgG were used as negative control. Graphs represent the percent of immunoprecipitated material relative to input DNA. (B) mRNA levels (real time qRT-PCR) of *pri-miR-101-2* in JR1 cells 48 h after EZH2 siRNA treatment were normalized to GAPDH levels and expressed as fold increase over CTR siRNA.
