# Supplementary material for: MicroRNA-101 is repressed by EZH2 and its restoration inhibits tumorigenic features in embryonal rhabdomyosarcoma
Source: Clin Epigenetics. 2015 Aug 6;7(1):82. doi: 10.1186/s13148-015-0107-z (PMC4527101; doi:10.1186/s13148-015-0107-z)
Supplement: Additional file 6: Table S1. — Clinical and histopathologic data of rhabdomyosarcoma tumor. [file 13148_2015_107_MOESM6_ESM.pdf]

Additional file 6: Table S1: Clinical and histopathologic data of Rhabdomyosarcoma tumor samples

Tumor Sample	*Diagnosis*	*Age (year)*	*Sex*	*Tumor status*	*Outcome*
1	ERMS	3	F	P	DOD
2	ERMS	1	M	P	Alive
3	ERMS	6	F	P	Alive
4	ERMS botryoid	4	F	P	Alive
5	ERMS	1	F	P	DOD
6	ERMS	4	M	P	Alive
7	ERMS	15	M	P	DOD
8	ERMS	12	M	P	Alive

Abbreviations: ERMS, embryonal rhabdomyosarcoma; P, primary; DOD, dead of disease.
